# Competitive RT-PCR Strategy for Quantitative Evaluation of the Expression of Tilapia (*Oreochromis niloticus*) Growth Hormone Receptor Type I

**DOI:** 10.1007/s12575-009-9002-3

**Published:** 2009-03-10

**Authors:** Alina Rodríguez-Mallon, Yamilet Cárdenas, Juana María Lugo, Aymé Oliva, Antonio Morales, Mario Pablo Estrada

**Affiliations:** 1 Aquatic Biotechnology Department, Animal Biotechnology Division, Center for Genetic Engineering and Biotechnology, P.O. Box 6162, Havana, 10 600, Cuba

**Keywords:** Tilapia, Receptors, Somatostatin

## Abstract

Quantization of gene expression requires that an accurate measurement of a specific transcript is made. In this paper, a quantitative reverse transcription-polymerase chain reaction (RT-PCR) by competition for tilapia growth hormone receptor type I is designed and validated. This experimental procedure was used to determine the abundance of growth hormone receptor type I transcript in different tilapia tissues. The results obtained with this developed competitive RT-PCR were similar to real-time PCR results reported recently. This protocol provides a reliable alternative, but less expensive than real-time PCR to quantify specific genes.

## 1. Introduction

Growth hormone (GH) plays a central role as a pluripotent endocrine regulator of physiological functions in fish and higher vertebrates, working through specific cell membrane receptor (GHR) that triggers a phosphorylation cascade for signaling and gene expression events [1,2]. The nucleotide sequence of GHR is available for mammals, birds, reptiles, and *Xenopus*. Based on conserved structural features, these receptors belong to class I cytokine receptor superfamily that include, among others, receptors for prolactin, erythropoietin, granulocyte colony stimulating factor, and several interleukins [3]. Since the initial cloning and sequence of goldfish (*Carassius auratus*) [4] and turbot (*Scophthalmus maximus*) [5] GHRs, other fish GHRs have been characterized in black sea bream (*Acanthopagrus schlegeli*) [6], gilthead sea bream (*Sparus aurata*) [7], masu salmon (*Oncorhynchus masou*) [8], rainbow trout (*Oncorhynchus mykiss*) [9], catfish (*Silurus meridionalis*), and tilapia (*Oreochromis niloticus*) [10]. Amino acid alignment of full-length GHRs reveals a relative high degree of identity (35–40%) among tetrapods and non-salmonid fish GHRs (GHR type I). Several authors have postulated a divergent evolution of salmonid GHRs (GHR type II); however, it has recently been cloned a GHR in rainbow trout (*Oncorhynchus mykiss*), which is analogous to GHRs of non-salmonid fish (GHR type I) and a GHR type II in non-salmonid fish [11]. Duplicated fish GHRs represent a new and perhaps complex step on the regulation of fish somatotropic axis. In this scenario, accurate measurements of both GHR expression patterns in different tissues and in different physiological stages are necessary. The classic methods to do this, such as Northern blots and RNAse protection assay, have been improved over the years and have provided reliable results. However, they share the weakness of having too low sensitivity among other drawbacks. Because of its extreme sensitivity, the polymerase chain reaction (PCR) has the potential to detect and precisely quantify specific RNA sequences if it is used in combination with reverse transcription. However, the repetitive multiplication of template molecules is a drawback for quantitative measurements because small differences in the multiplication factor lead to large differences in the amount of product [12]. Although the use of PCR for quantification has been uncritically accepted by many scientists, it really cannot be relied upon for quantitative measurements. Two methods can be used to solve the problem of quantification: kinetic methods and co-amplification methods. Co-amplification methods can be done without expensive equipment. In this study, we design a competitor molecule to quantify accurately the tilapia growth hormone receptor type I (tiGHR I) in different tilapia tissues using a quantitative RT-PCR by competition and we show that it is sensitive, reproducible, and robust.

## 2. Materials and Methods

### 2.1. Cloning a TiGHR I Probe

We designed four forward degenerate oligonucleotides (A, B, C, and D) that contained all the coding sequences for a conserved N glycosilation site of the GHR extracellular domain (LNWTLLNI) and four reverse degenerate oligonucleotides (E, F, G, and H) that contained all the coding sequences corresponding to the proline-rich site in the intracellular domain box I (PKIKGIDP) (Table 1).

**Table 1:** Forward and reverse degenerate oligonucleotides to clone tiGHR I probe

Oligonucleotides	Sequences
A	5'...(tc)t(ag)aa(ct)tggac(acgt)(tc)t(ag)tt(ag)aa(ct)at...3'
B	5'...ct(tc)aa(ct)tggac(acgt)tt(ag)(ct)t(ag)aa(ct)at...3'
C	5'...(ct)t(ag)aa(ct)tggac(acgt)ct(tc)ct(tcag)aa(tc)at...3'
D	5'...ct(tc)aa(ct)tggac(acgt)ct(tcag)ct(tc)aa(tc)at...3'
E	5'...gg(ga)tc(tag)at(tg)cc(tc)tt(tga)at(tc)tt(tg)gg...3'
F	5'...gg(ga)tc(tga)at(tg)cc(tc)tt(tga)at(tc)tt(ca)gg...3'
G	5'...gg(ga)tc(tag)at(ca)cc(tc)tt(tga)at(tc)tt(tg)gg...3'
H	5'...gg(ag)tc(tag)at(ca)cc(tc)tt(tga)at(tc)tt(ca)gg...3'

Total RNA from tilapia liver (*O. niloticus*) was obtained by the acid phenol method [13]. Messenger RNA was purified from total RNA using the "PolyAtract^®^ mRNA Isolation System III" kit (Promega, USA). Messenger RNA was reverse transcribed with oligo (dT) 15 using "Reverse Transcription System" (Promega). Polymerase chain reactions were set up in 50-μl volumes using "PCR Master Mix" (Promega) with 3 μM of forward and reverse primers and 1/10 volume of the RT reaction. We used all possible combinations of the degenerated oligonucleotides (16 different reactions). The PCR condition used 95°C for 3 min, followed by a cycling program of 94°C for 1 min, 42°C for 1 min and 72°C for 1 min for 30 cycles, and a final extension at 72°C for 5 min. PCR products were purified from agarose gel using "Qiaquick^®^ Gel Extraction" Kit (Qiagen, USA) and cloned in T-vector (pGEM^®^-T Easy Vector System I, Promega).The selected clones were sequenced using standard techniques [14].

### 2.2. Generation of Competitor

**Figure 1:**
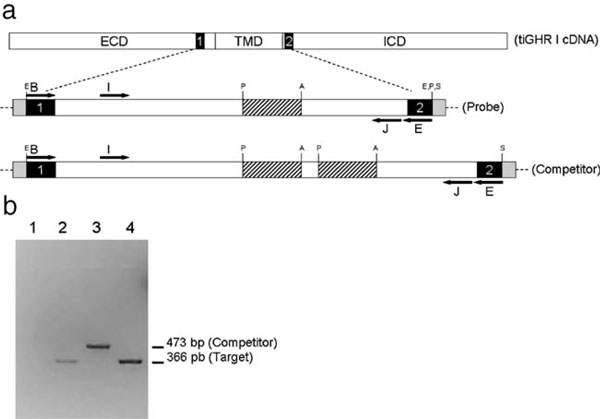
**Design for the isolation of probe from the tiGHR I cDNA and construction of competitor fragment to use in quantitative PCR**. **a** Diagram of probe amplification from the tiGHR I cDNA using degenerate oligonucleotide B and E. *ECD*, extracellular domain; *TMD*, transmembrane domain; *ICD*, intracellular domain; *black box 1*, conserved N glycosilation site of the GHR extracellular domain (LNWTLLNI); *black box 2*, coding sequences corresponding to the proline-rich site in the intracellular domain box I (PKIKGIDP); *Probe*, 458-bp DNA fragment of tiGHR I obtained with the B–E oligonucleotide mixes and cloned in Easy T-vector (*shadow boxes*); *Competitor*, 473 DNA fragment obtained from two subcloning steps to produce a tandem of two copies of the Pst I–Acc I fragment of the Probe; *I* and *J*, internal oligonucleotide from the probe fragment designed for the amplification of target and competitor; *E*, EcoR I; *P*, Pst I; *A*, Acc I; *S*, Sac I. **b** 2% agarose gel image with the PCR amplification products using oligonucleotides I and J: *1*, PCR negative control (without template); *2*, PCR using a RT reaction from tilapia liver mRNA as template; *3*, PCR using competitor sequence as template, *4*, PCR using probe as template.

Starting with a clone containing an insert of 458 bp of tiGHR I (Probe), we did two subcloning steps to obtain an internal duplication of 100 bp respect to original fragment, generating a fragment used as competitor (Figure 1). The competitor was linearized with the endonuclease Sma I and transcribed in vitro using "T7 RiboMAX Express RNAi System" kit (Promega) to obtain the competitor RNA.

### 2.3. Competitive PCR

We designed two specific oligonucleotides (I = ccccacctactgctgatgttag and J = caggaacaggcggcagcagg) that hybridize inside to the fragment of the tiGHR gene cloned between binding sites of degenerate oligonucleotides. When we use these specific oligonucleotides in a PCR, we generate a 366 bp amplification product from the wild-type DNA (T-target) and a 473 bp amplification product from the competitor DNA (Ccompetitor) (Figure 1b). The PCR reactions were set up in 50 μl with "PCR Master Mix" and 0.2 μM of each primer. We used a denaturalization step of 95°C for 2 min, followed by a cycling program of 94°C for 30 s, 62°C for 30 s, and 72°C for 1 min for 30 cycles. A PCR negative control was set up for all the PCR batches to ascertain the authenticity of PCR. The amplification products were resolved in 2% agarose gels with ethidium bromide. Gel images were obtained using a digital camera Olympus C7070 Wide Zoom. The photos saved in jpeg format were used for densitometry analysis.

### 2.4. Quantitative Analysis

The densitometry data for band intensities in different sets of experiments was generated by analyzing the gel images on the Image J program (Version 1.33, USA). Previously to the experiments of competitive PCR, we did an experiment (data not shown) to control the consistency of our densitometry raw data. Because of the low dynamic range of ethidium bromide gels, it is necessary to control if the peak areas corresponding to densitometry values obtained by Image J program reproduce really the band intensities. In this experiment, we used a wide range of concentration of DNA and considered the relation dose–response. The lineal relation is lost after 100 ng of DNA.

### 2.5. Determination of Target/competitor Amplification Efficiency

Ten identical PCR mixtures were prepared, as described above, each containing 100,000 molecules of target and competitor DNA. The PCR cycling conditions were carried through 45 cycles with one tube being removed after 17, 19, 21, 24, 27, 30, 33, 36, 39, and 45 cycles and the amount of the PCR products quantified. Procedure was repeated two times. Efficiency was calculated as Ei = (Pi - Pi - 1)/Pi - 1 [12], where Ei is the efficiency in one step, Pi is the quantity of product in that step, and Pi - 1 is the product already accumulated during the previous step. The efficiency means for target and competitor in each cycle were compared using matched *t* test.

### 2.6. Determination of Accuracy of the Competitive PCR

To test the precision of the results obtained with this competitive PCR, five different amounts of T (10,000, 100,000, 150,000, 200,000, and 1,000,000 molecules) were assayed with serial dilutions of the C. Each set of validation experiments comprised at least four reaction combinations (T related to C) with three replicas for each point in the conditions described above. One of these experiments (100,000 molecules of target with six competitor dilutions with three replicas of each point) was repeated three times in different days. These produced a data set of 120 reactions to address the intra- and inter-experiment variability, precision, and resolution of our experimental system.

### 2.7. Sensitivity of Competitive PCR

To test the minimum quantity of target molecules that our PCR is able to detect, we did five sets of experiments ranging from 2,000 until 10 molecules of target with three different dilutions of competitor with two replicas for each point in the conditions set up above.

### 2.8. Competitive RT-PCR

To determine the tiGHR I expression levels in different tilapia tissues, we started from total RNA mini-preparations of each tissue using the acid phenol method [13]. We used three juvenile tilapias (*O. niloticus*) of 100 g as source of 100 mg of tissue from liver, muscle, brain, heart, stomach, spleen, intestine, and gonads. The RT-PCR reactions were done using "Ready-To-Go™ RT-PCR Beads" (Amersham Biosciences, USA) using the same cycling profile described before. For each sample of total RNA, we used at least two known different quantities of competitor RNA molecules to obtain linear regressions. We determined the number of target molecules in the sample when log (T/C) equals zero [15]. In this way, the target molecules number for all tissue samples of each tilapia was obtained. These values were normalized versus total RNA (RNAt) used to do the RT reaction. The same quantity of RNAt used in the RT reaction was electrophoresed on 1.5% formaldehyde agarose gel. The densitometry data of the bands corresponding to the 28S subunits measured with Image J program were converted to micrograms of RNAt using a reference RNAt with known concentration. Then, the results were expressed as number of tiGHR I molecules/μg RNAt. The obtained averages of the tiGHR I molecules/μg RNAt for each tissue were compared by non-parametric Kruskal–Wallis test followed by Dunn multiple comparison test (Prism, version 4.0 for Windows; GraphPad Software, USA).

## 3. Results

### 3.1. Cloning TiGHR I Probe, Construction of Competitor, and Verification of Differentiable Amplification

**Figure 2:**
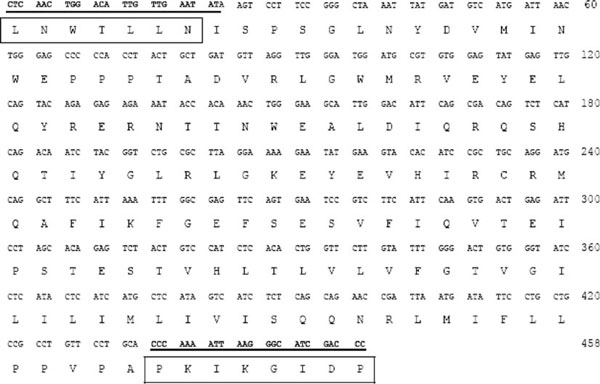
**Sequence of the probe corresponding to a 458-bp fragment of tiGHR I**. In *bold* and *underline*, oligonucleotides corresponding to mix B and E, respectively; *inside box*, amino acid sequences used to design degenerated oligonucleotides.

The combination between the four forward degenerated oligonucleotides (A, B, C, and D) and the four reverse degenerated oligonucleotides (E, F, G, and H) give 16 different PCRs. The bands nearby 500 bp were cloned in T-vector and sequenced. One clone (probe) with a 458 bp fragment of tiGHR I was obtained with the B–E oligonucleotide mixes (Figure 2). This fragment is 100% identical to the reported GHR type I from tilapia (*O. niloticus*) (accession number AY973232), without the sequence of the degenerated oligonucleotides. After the competitor construction, we were able to see a difference in the electrophoretic mobility between the amplification products of wild-type and competitor sequences of tiGHR I using I and J oligonucleotides in the PCR (Figure 1b).

### 3.2. Verification of Equal Amplification Efficiencies for Target and Competitor

**Figure 3:**
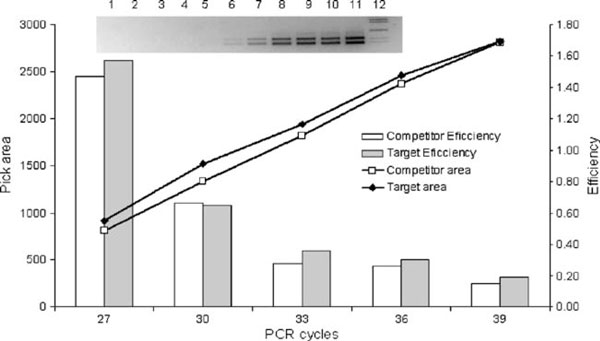
**PCR efficiencies of target and competitor**. In the picture: *1*, PCR negative control; amplification products at 17, 19, 21, 24, 27, 30, 33, 36, 39, and 45 cycles corresponding to lines *2*, *3*, *4*, *5*, *6*, *7*, *8*, *9*, *10*, and *11*, respectively, using 10^5^ molecules of competitor and target in the PCR; *12*, molecular weight marker (from *top* to *bottom* = 1.4 kb, 1.2 kb, 750 bp, 450 bp, and 366 bp). *Lines* represent densitometry data of the target and competitor band intensities versus cycle number. Each *point* is the average between two different experiments. *Bars* represent the target and competitor PCR efficiencies for each cycle. There are no statistically significant differences between target and competitor efficiencies in any cycles determined using matched *t* test (*p* > 0.05).

Target and competitor amplify in the competitive PCR with the same efficiency over 24–39 cycles (Figure 3). No statistically significant differences between the cycle efficiency averages of the competitor and target sequences were encountered using a paired *t* test (*p* < 0.05). All subsequent competitive PCRs were carried out for 30 cycles, when the amplification was found to be lineal and the efficiency was diminishing but it was equal for the target and competitor sequences. In addition, to 30 cycles the quantity of amplification products in our PCR conditions is sufficiently visible in an ethidium bromide gel and keeps the lineal relationship to measured peak areas. It means that the band intensities are far from the gel saturation point.

### 3.3. Accuracy of Competitive PCR

**Figure 4:**
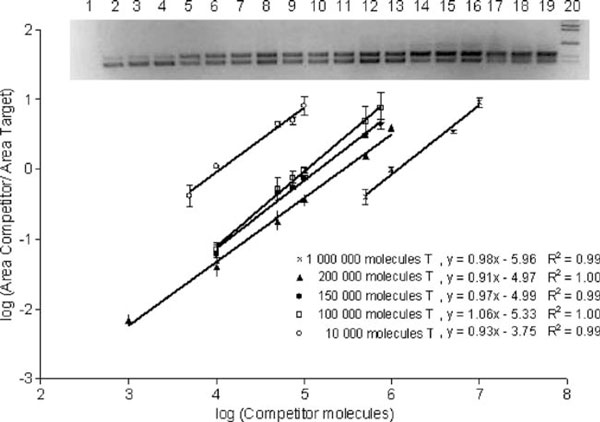
**Lineal regressions generated from the densitometry data of the PCR reaction using fixed target molecule numbers and serial dilutions of competitor**. In the picture, typical 2% gel image of amplification products in the competitive PCRs where 10^5^ molecules of target were used with serial dilutions of competitor. *1*, PCR negative control; *lanes 2*–*4*, 10^4^; *lanes 5*–*7*, 5 × 10^4^; *lanes 8*–*10*, 7.5 × 10^4^; *lanes 11*–*13*, 10^5^; *lanes 14*–*16*, 5 × 10^5^; *lanes 17*–*19*, 7.5 × 10^5^ molecules of competitor, respectively; *20*, molecular weight marker (from *top* to *bottom* = 1.4 kb, 1.2 kb, 750 bp, 450 bp, and 366 bp).

The raw data collection of the 120 amplification reactions is given in file 1 of the Electronic supplementary material. Five different quantities of the target sequence (10,000, 100,000, 150,000, 200,000, and 1,000,000 molecules) were amplified in the presence of dilution series of the competitor sequence. The log ratio of the quantity of products versus the log of the competitor sequence added was plotted (Figure 4). The validity of the competition reaction data at different target concentrations is represented by regression equations with respective *R*^2^ values. Each point in the best fitted regression line is the average among three replicas. The calculated concentration of the target sequence is equal to the concentration of competitor sequence, determined from the regression line, when the log ratio of the products equals zero. There is a good correlation between the observed and expected concentrations of the target sequence characterized by a Pearson *r* of 0.9978 (95% CI 0.9656–0.9999) with *p* < 0.001 extremely significant (Table 2). Intra- and inter-experiment repeatability was measured. The coefficients of variation among the replicas of the experimentally determined target concentrations in each experiment are shown in Table 3. The experiment using 100,000 molecules of target sequence with six different dilutions of the competitor sequence with three replicas by each point was repeated three times in different days. Triplicate analyses yielded an experimentally determined quantity of target of 107,000 + 8,622 molecules with an inter-experiment coefficient of variation of 8%.

**Table 2:** Number of the expected and observed target molecules

**Number of target molecules**^**a**^	
**Expected**	**Observed**

10,000	11,000
100,000	107,000
150,000	146,000
200,000	290,000
1,000,000	1,180,000

^a^Characterized by a Pearson *r* of 0.9978 (95% CI 0.9656–0.9999) with *p* < 0.001

**Table 3:** Intra-experiment variability

				**100,000**^**a**^			
					
Expected molecules target	1,000,000	200,000	150,000	A	B	C	10,000
Observed molecules target^b^	1,173,333	290,000	255,000	115,000	110,667	97,333	11,200
SD	161,658	35,000	91,788	5,000	9,292	3,055	2,081
CV	13.78%	12.07%	36%	4.35%	8.40%	3.14%	21.89%

^a^Experiments A, B, and C correspond to experiments developed on three different days

^b^Each value corresponds to the mean of three experimental replicates

### 3.4. Sensitivity of Competitive PCR

Seven hundred and fifty molecules in 50 μl of PCR is the lower limit of quantification (LLQ) of target DNA sequence that the competitive PCR was able to detect.

### 3.5. Competitive RT-PCR in Different Tilapia Tissues

**Figure 5:**
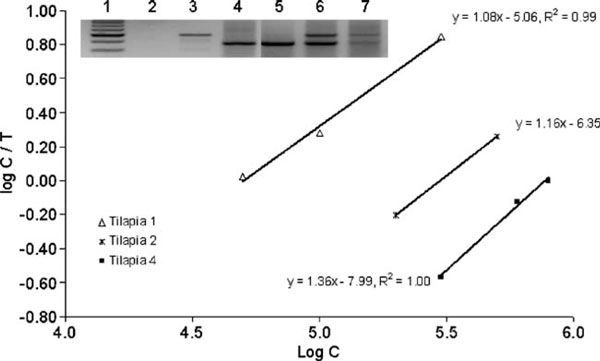
**Determination of the quantity of tiGHR I RNA in tilapia liver**. Lineal regressions with data from three different tilapias. The picture is an example of 2% agarose gel with the amplification products of competitive RT-PCR using total RNA from the liver of tilapia 4 and known quantities of competitor RNA. *1*, molecular weight marker (leader 100 bp, Promega); *2*, RT-PCR negative control; *3*, RT-PCR with 5 × 10^5^ molecules of RNA competitor alone; *4*, RT-PCR with liver total RNA of tilapia alone; *5*, *6*, and *7*, RT-PCR of liver total RNA of tilapia 4 with 3 × 10^5^, 6 × 10^5^, and 8 × 10^5^ molecules of competitor, respectively.

**Figure 6:**
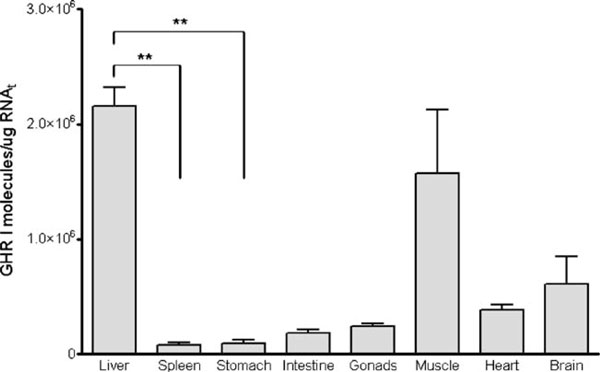
**Expression of tiGHR I in different tilapia tissues**. The non-parametric test of Kruskal–Wallis followed by Dunn's post test only detected significant differences between liver and spleen and between liver and stomachs (*p* < 0.05).

Abundance levels of tiGHR I mRNA (target) in different tilapia tissues were measured using the quantitative RT-PCR by competition validated above. Liver RNA of three different tilapias was assayed in the presence of different quantities of competitor RNA. Densitometry data derived from band intensities of competitor and target for each tilapia were plotted as described in Section 2 (Figure 5). We discarded the amplification of genomic DNA sequences because the specific oligonucleotides I and J hybridize to different exons, which means that, in the genomic DNA sequences between these oligonucleotides, there are introns. PCR amplifications from genomic DNA sequences would give a higher size than 366 bp and 473 bp expected for target and competitor amplifications, respectively. The quantity of specific RNA obtained for each tilapia liver was normalized versus the band intensity of the 28S subunit of the total RNA used in the RT reaction. The quantity of tiGHR I RNA reported here for liver is the average of the normalized data obtained from each tilapia. The coefficient of variation is presented in the Electronic supplementary material 2. The procedure with the other tilapia tissues was the same. The raw data can be seen too in file 2 of the Electronic supplementary material. The coefficients of variation of these experiments were between 10% and 50%. After the processing of all obtained data from the selected tilapia tissues, we obtained the profile of the expression levels of tiGHR I mRNA (Figure 6).

## 4. Discussion

The exponential character of PCR amplification may compromise quantitative assays because it multiplies variations. The competitive PCR strategy used in the present study was aimed to overcome some of the limitations of the conventional RT-PCR. Co-amplification methods quantify the target DNA relative to a second control sequence in the same PCR tube. The main advantages of this technique are that the results are not affected by tube-to-tube variations in amplification efficiency and it is not necessary to restrict PCR to the exponential phase. Reliable quantification is still possible if the PCR extends into the linear phase, or even in the saturation phase, provided it is ascertained that the amplification efficiency is the same for both templates throughout the PCR, including the final cycles [12,16]. Quantitative co-amplification rests on the assumption that the product ratio of target and competitor sequences reliably reflects the ratio of their initial copy numbers. Therefore, it is requisite that efficiency is identical for both sequences. If the target sequence (T) and the competitor sequence (C) would amplify with the smallest difference in this efficiencies, it can lead to very different quantities of the end products. This can result in an erroneous estimation of the amount of initial material [12,16]. Figure 3 shows that the efficiencies in our system for target and competitor are equal in each cycle, even if the efficiencies decrease in the later cycles. The validity of the competition reactions to each quantity of target was established by the generated regression equations with their corresponding significant *R*^2^ values (Figure 4). The slope of the regression curves obtained was close to 1 in all five standard curves, indicating that no differential amplification rates exist between T and C in the range of assayed T. Variability in the 10,000- to 1,000,000-molecule ranges of target sequence was determined. Coefficients of variation according to non log-transformed absolute values were 8% inter-assay and ranging between 3.14% and 8.4 % intra-assay for 100,000 molecules of T. CVs of 21.89%, 36%, 12.07%, and 13.78% intra-assay for 10,000, 150,000, 200,000, and 1,000,000 molecules of T, respectively were obtained (Table 3). In general, lower target quantities give higher CVs. Our variability is in the range of the variability of the PCR assay [17,18]. In general, CVs corresponding to reaction conditions, in which the quantity of target and competitor was equivalent or nearby, were under 10%, and CVs corresponding to reaction conditions, in which the ratio C/T was 10 or 1/10, were between 10% and 35%. Other authors affirm that more time-consuming methods of RNAse protection assay and Northern blots are more accurate and precise than RT-PCR. However, it has been reported that RNAse protection assay could detect approximately 1 × 106 target transcripts and it has been estimated that a Northern blot is about tenfold less sensitive than RNAse protection assay [19], then they are limited to study those genes that are relatively highly expressed. Our competitive PCR was able to detect at least 750 molecules of DNA target sequence in 50 μl of PCR. If we assume 10% of efficiency of the RT reaction, the LLQ of our system would be 7.5 × 10^3^ target transcripts. Therefore, this assay is more sensitive than RNase protection and Northern blot assays. Our results show that our design of competitive PCR together with RT reaction is useful to study the expression level of tiGHR I in different tissues of tilapia (*O. niloticus*). By using in vitro transcribed competitor RNA, we have been able to reduce sources of variation such as the variable efficiency of the reverse transcription reaction because the quantity of competitor RNA molecules that we put together with specific tissue total RNA samples is reverse transcribed with the same efficiency that the molecules of RNA target in each sample.

We have also been able to detect expression of this receptor in all studied tissues, which is consistent with the pleiotropic nature of growth hormone in fish [20-23]. The expression level of tiGHR that we obtained for each studied tissue can be organized in decreased order of expression levels as: liver > muscle > brain > heart > gonads > intestine > stomach > spleen (Figure 6). The highest expression level of tiGHR I in liver is consistent with previous receptor binding studies [20]. Besides, it is in agreement with previous conventional RT-PCR studies [4,6] and with real-time PCR studies [24]. As it is expected, the tissue distribution obtained for us is more similar to the real-time RT-PCR results than to the results of the other studies using conventional RT-PCR. The fact that we observed statistically significant differences only between liver–spleen and between liver–stomachs is due to a non-parametric test that we used. These tests are less powerful than the parametric tests that assume data Gaussian distributions. With small samples (*n* = 3), non-parametric tests have little power to detect differences especially when we work with biological samples that are intrinsically variable. In future experiments, we will work with a higher number of animals. To refine our initially found results, we would retest them with a higher resolution of competitor molecules because we demonstrate in the validation experiments that the coefficients of variability are lower when the molecule numbers of target and competitor sequences in the samples are similar.

Despite the fact that the described competitive RT-PCR assay is labor intensive, less sensitive, and has a lower dynamic range than the real-time assays, it is less expensive than the real-time RT-PCR studies.

In summary, through this work, we have developed a quantitative RT-PCR assay by competition that was sensitive enough to differentiate among mRNA abundance levels of tiGHR I. The nature of competition reactions observed was supportive to prove the authenticity of quantification of tiGHR I.

## Abbreviations

Ab: Antibiotic; C: Competitor; DNA: Deoxyribonucleic acid; GH: Growth hormone; GHR: Growth hormone receptor; mRNA: Messenger ribonucleic acid; o.n.: Overnight; PCR: Polymerase chain reaction; RNA: Ribonucleic acid; RT: Reverse transcription; T: Target; tiGHR I: Tilapia growth hormone receptor type I; *T*_m_: Melting temperature

## Appendix

### PROTOCOLS

I- Cloning of competitor

   *Materials*

• RNAgents: Total RNA Isolation System, Promega Z5110

• PolyATract: mRNA Isolation System II, Promega Z5200

• Reverse Transcription System, Promega A3500

• PCR Master Mix, Promega M7505

• QIAquick Gel Extraction Kit, QIAGEN 28704

• pGEM^®^-T Easy Vector System I, Promega A1360 and pBluescript^®^ II Phagemid Vectors, Stratagene 212207 to cloning DNA sequences

• Reagents for cloning

• Ampicillin and streptomycin for selection purposes

• Degenerated primers to amplify tiGHR I probe sequence

• Top 10 (F^-^ mcrA Δ[mrr-hsdRMS-mcrBC] ø80 lacZ Δ M15 Δ lacX74deoR recA1 araD139 Δ[ara-leu] 7697 galV galK rpsL [StrR] endA1nupG) or equivalent electrocompetent *E. coli* cells

• T7 RiboMAX TM Express RNAi System, Promega P1700

• MEGAscript^®^ RNAi Kit, Ambion 1626

   *Methods*

1. To obtain total RNA of tilapia (*O. niloticus*) liver, we followed the procedure described in the section IV of the Technical Bulletin 087 (TB087) of RNAgents^®^ Total RNA Isolation System (Promega). We started with 1 g of tilapia liver.

2. Starting with 5 mg of tilapia liver total RNA, we obtained mRNA following exactly the protocol described in the section IV in the Technical Manual 021 (TM 021, Promega) to PolyAtract^®^ Systems I.

3. RT-PCR amplifies the tiGHR I probe. The reverse transcription reaction was performed with 1 μg of tilapia liver mRNA and Oligo (dT)_15_ as primer following the protocol described in the section III of the Technical Bulletin 099 (TB099) of Reverse Transcription System (Promega, USA). We used 10 μl of the five times diluted RT reaction in 50 μl of the PCR final volume. We used too 25 μl of PCR Master Mix 2× (Promega) and 3 μM of each degenerated primer (150 pmol/50 μl PCR). We performed 3 min to 95°C to denaturalize all DNAs in the reaction and after we did cycling program (1 min to 94°C, 1 min to 42°C, and 1 min to 72°C) for 30 cycles and a final extension 5 min to 72°C.

4. Clone the PCR product nearby to 500 bp into the pGEM-T Easy, or equivalent, vector and transform into electrocompetent top ten *E. coli* cells, or equivalent, for sequence verification.

5. Sub-clone the 3' region of the tiGHR I probe into pBS KS + vector and ensemble again duplicating an internal Pst I–Acc I fragment (Figure 1a).

6. Linearize the plasmid that contains the competitor sequence under the control of T7 RNA polymerase promoter with appropriated restriction enzyme.

7. Synthesize single-stranded transcript of competitor RNA. We followed the protocol described in the section III-C of the Technical Bulletin 316 of the T7 RiboMax™ Express RNAi System (Promega)

8. Remove the DNA template by digestion with RNase-free DNase of the T7 RiboMax™ Express RNAi System.

9. Purification of ssRNA was carried out following the protocol described in the section III-E of the Instruction Manual of the MEGAscript^®^ RNAi Kit (Ambion, USA).

10. Quantitate the product by measuring its absorbance at 260 nm and examine the integrity on a 1% denaturing agarose gel.

11. Store RNA competitor precipitated in EtOH at -20°C.

II. Validation of Competitive PCR

   *Materials*

• Linear DNA of target and competitor sequences.

• Gene-specific primers to amplify target and competitor sequence.

• PCR Master Mix, Promega M7505

• Reagents and equipment to do DNA electrophoresis.

• Digital camera Olympus C7070 Wide Zoom

• Image J program (Version 1.33).

   *Methods*

**Figure 7:**
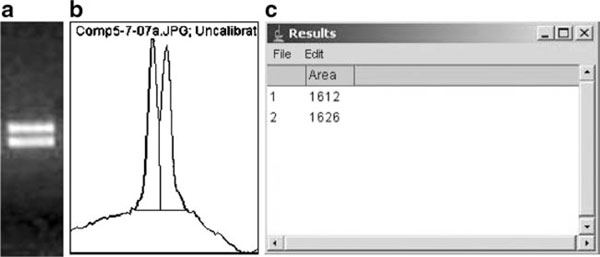
**Densitometry analysis of competitor and target bands using Image J Program**. **a** User-defined selection from 2% agarose gel digital image. **b** Image J plots of selected image. **c** The area measurements displayed in a Results window by the image J Program.

1. Determination of equal PCR amplification efficiency of target and competitor sequences.

a. Ensemble ten identical PCR tubes containing equimolecular quantities of target and competitor sequences in 50 μl as final volume. Use 0.2 μM of each specific primer and 25 μl of PCR Master Mix. Perform 3 min to 95°C to denature all DNA and a cycling program 30 s to 94°C, 30 s to annealing temperature according the Tm of the specific oligonucleotides, and 1 min to 72°C. Remove one tube at 17, 19, 21, 24, 27, 30, 33, 36, 39, and 45 cycles, respectively, and store at -20°C.

b. Electrophoresis in 2% agarose gel in TA 1× (0.04 M Tris–acetate, 0.001 M EDTA, pH 7) of the amplification products of each tube.

c. Take the digital images of the gel.

d. Densitometry analysis of competitor and target bands in each lane using Image J Program (Version 1.33). This program is ideal to compare bands in the same digital image. It is based on the fact that optical density (OD) is a logarithmic function of brightness. A set of macros are bundled to Image J which are used for gel densitometry analysis producing curves. The height of the curve, at any given point, is the mean of the OD of a given row of pixels in the marked lane. The program can calculate area of user-defined selections. The area measurements are recorded in tabular form and are displayed in a Results window as shown in Figure 7. To get more information, visit http://rsb.info.nih.gov/ij/

e. Plot the area measurements versus cycle number to target and competitor, respectively (Figure 3).

f. Calculate efficiency to each cycle to target and competitor as was described in Section 2.

g. Compare the efficiencies of target and competitor using a paired t test.

h. According to the results, establish 30 cycles for all competitive PCRs.

2. Determination of the sensibility of this method.

a. Ensemble PCRs in the same conditions established before using decreased quantities of target with different dilutions of competitor with replicas for each point. In our experiment, we ensemble:

• 2,000 molecules of target with 750, 1,000, and 5,000 molecules of competitor, respectively.

• 1,000 molecules of target with 500, 1,000, and 5,000 molecules of competitor, respectively.

• 750 molecules of target with, 250, 750, and 1,000 molecules of competitor, respectively.

• 100 molecules of target with 50, 100, and 500 molecules of competitor, respectively.

• 10 molecules of target with 5, 10, and 50 molecules of competitor, respectively.

b. Electrophoresis in 2% agarose gel in TA 1× (0.04 M Tris–acetate, 0.001 M EDTA, pH 7) of the amplification products of each tube.

c. Determination of minimum quantity of target that the method is able to detect.

3. Characterization of the method precision and repeatability.

a. Ensemble PCRs in the same conditions established before using quantities of target in the range of your expected measurements with different dilutions of competitor with replicas for each point. In our experiment, we ensemble:

• 10^4^ molecules of T with 5 × 10^3^, 10^4^, 5 × 10^4^, 7.5 × 10^4^, and 10^5^ molecules of C, respectively, with three replicas for each point.

• 10^5^ molecules of T with 10^4^, 5 × 10^4^, 7.5 × 10^4^, 10^5^, 5 × 10^5^, and 7.5 × 10^5^ molecules of C, respectively, with three replicas for each point. This experiment was repeated three times in different days to assay the inter-experiment variability.

• 1.5 × 10^5^ molecules of T with the same molecule quantities of C as the previous experiment with three replicas for each point.

• 2 × 10^5^ molecules of T with 10^3^, 10^4^, 5 × 10^4^, 10^5^, 5 × 10^5^, and 10^6^ molecules of C, respectively, with three replicas for each point.

• 10^6^ molecules of T with 5 × 10^5^, 10^6^, 5 × 10^6^, and 10^7^ of C, respectively, with three replicas for each point.

b. Electrophoresis in 2% agarose gel in TA 1× (0.04 M Tris–acetate, 0.001 M EDTA, pH 7) of the amplification products of each tube.

c. Take digital images of the gel.

d. Densitometry analysis of competitor and target bands in each lane using Image J Program (Version 1.33) as was described in point 1d of this section.

e. Make lineal regressions for each experiment plotting log of the ratio between the area measurements of C and T versus log of the C molecule numbers. Determine the equation of each regression with its respective R2.

f. Determine the x value (C molecule number) when y = 0. This condition occurs when the area measurements of C and T are equal because C/T = 1 and the log1 = 0. Then, x value is equal to the calculated T molecular number in each experiment.

g. Plot T molecule numbers expected versus observed and determine the correlation coefficient (r). We used the test of Pearson correlation. This determination represents a measure of the precision of our method in the range of the T quantities assayed.

h. Calculate the variation coefficients (CVs) intra-experiment using the replicas inside experiments and inter-experiments. These CV values characterize the repeatability of our method in the range of the T quantities assayed.

III. Competitive RT-PCR

   *Materials*

• RNAgents: Total RNA Isolation System, Promega Z5110

• Competitor RNA generated in the Protocol I

• Gene-specific primers to amplify target and competitor sequence.

• Ready-to-Go RT-PCR Beads, Amersham Biosciences, 27-9259-01

• Reagents and equipment to do DNA electrophoresis.

• Digital camera Olympus C7070 Wide Zoom

• Image J program (Version 1.33).

   *Methods*

1. Isolate total RNA of eight different tissues (brain, muscle, heart, liver, gonads, stomach, spleen, and intestine) from three tilapias (O. niloticus). Total RNA mini-preparations starting with 50 mg of each tissue were performed using RNAgents. Total RNA Isolation System, Promega. (see table 1 of the TB087).

2. Ensemble reactions of RT-PCR using Ready-to-Go RT-PCR Beads, Amersham Biosciences. We used 2 μl of each preparation of total tissue-specific RNA with at least three known different quantities of competitor RNA and random primers to the RT reactions and 0.2 μM of each specific primer to the PCR.

3. Electrophoresis in 2% agarose gel in TA 1× (0.04 M Tris–acetate, 0.001 M EDTA, pH 7) of the amplification products of each tube.

4. Take digital images of the gel.

5. Densitometry analysis of competitor and target bands in each lane using Image J Program (Version 1.33) as was described in point 1d of this section.

6. Do lineal regressions for each experiment plotting log of the ratio between the area measurements of C and T versus log of the C molecule numbers. Determine the equation of each regression.

7. Determine the x value (C molecule number) when y is equal to 0. In this condition, x value is equal to T molecule number in each RNA sample.

8. Normalize this T molecule number in each RNA sample with the input of total RNA in the RT reaction. The total RNA input was determined by densitometry analysis of 28S subunit of ribosomal RNA in each sample using Image J program. We did an electrophoresis on 1.2% agarose denaturalizing gel of 2 μl of each total RNA preparation. The densitometry data of the bands corresponding to the 28S subunits measured with Image J program were converted to micrograms of total RNA using a reference RNA with known concentration. Then, the results were expressed as tiGHR I molecule numbers/μg RNAt.

9. Average the tiGHR I molecule numbers/μg RNAt among the three tilapias to each tissue. These averages were compared by non-parametric Kruskal–Wallis test followed by Dunn multiple comparison test.

## Supplemental Information

The raw data collection of the 120 amplification reactions performed to validate our competitive RT-PCR is given in supplementary data file 1. The raw data of the determination of the tiGHR I levels in each tilapia tissue and their coefficients of variation can be seen in the supplementary data file 2.

## Supplementary Material

Additional file 1 Click here for file

Additional file 2 Click here for file
