# Supplementary material for: Competitive RT-PCR Strategy for Quantitative Evaluation of the Expression of Tilapia (Oreochromis niloticus) Growth Hormone Receptor Type I
Source: Biol Proced Online. 2009 Mar 10;11:79–98. doi: 10.1007/s12575-009-9002-3 (PMC3055623; doi:10.1007/s12575-009-9002-3)
Supplement: Additional file 1 [file 1480-9222-11-1-9002-S1.pdf]

Supplemental file 1

Target Molecules (T)	Competitor Molecules (c)	log c		Pick Area (A)	Ac/AT	log(Ac/AT)	calculated X	calculated competitor molecules	Averages of log (C/T)	SD
200 000	1000	3.0	C	21	8.0E-03	-2.10	3.36	2300	-2.17	0.09
			T	2633						
			C	20	7.5E-03	-2.13	3.33	2150		
			T	2683						
			C	12	5.4E-03	-2.27	3.19	1560		
			T	2242						
	10000	4.0	C	113	6.0E-02	-1.22	4.24	17500	-1.39	0.15
			T	1851						
			C	58	3.6E-02	-1.44	4.01	10200		
			T	1620						
			C	37	3.1E-02	-1.51	3.95	9000		
			T	1189						
	50000	4.7	C	272	2.3E-01	-0.64	4.82	66000	-0.73	0.14
			T	1190						
			C	138	1.3E-01	-0.90	4.56	36500		
			T	1093						
			C	194	2.2E-01	-0.66	4.80	63000		
			T	882						
	100000	5.0	C	450	4.5E-01	-0.34	5.11	130000	-0.45	0.09
			T	994						
			C	290	3.1E-01	-0.50	4.95	90000		
			T	925						
			C	334	3.3E-01	-0.49	4.97	94000		
			T	1026						
	500000	5.7	C	784	1.5E+00	0.17	5.63	430000	0.18	0.04
			T	527						
			C	731	1.4E+00	0.15	5.60	400000		
			T	522						
			C	745	1.7E+00	0.23	5.69	490000		
			T	426						
	1000000	6.0	C	808	3.5E+00	0.55	6.01	1025000	0.59	0.04
			T	228						
			C	802	4.0E+00	0.60	6.06	1150000		
			T	202						
			C	937	4.2E+00	0.62	6.08	1200000		
			T	224						

Target Molecules (T)	Competitor Molecules (c)	log c		Pick Area (A)	Ac/AT	log(Ac/AT)	calculated X	calculated competitor molecules	Averages of log (C/T)	SD
150 000	10000	4.0	C	73	4.4E-02	-1.36	3.81	6500	-1.21	0.13
			T	1672						
			C	121	7.1E-02	-1.15	4.02	10500		
			T	1696						
			C	126	7.4E-02	-1.13	4.04	11000		
			T	1701						
	50000	4.7	C	589	4.5E-01	-0.35	4.82	66000	-0.35	0.01
			T	1309						
			C	587	4.4E-01	-0.36	4.80	63000		
			T	1349						
			C	614	4.6E-01	-0.34	4.83	68000		
			T	1332						
	75000	4.9	C	686	5.5E-01	-0.26	4.91	81000	-0.26	0.02
			T	1241						
			C	610	5.2E-01	-0.28	4.88	76000		
			T	1172						
			C	671	5.7E-01	-0.25	4.92	83000		
			T	1182						
	100000	5.0	C	789	7.8E-01	-0.11	5.06	115000	-0.13	0.03
			T	1008						
			C	785	7.7E-01	-0.12	5.05	113000		
			T	1026						
			C	716	7.0E-01	-0.16	5.01	102000		
			T	1026						
	500000	5.7	C	1289	3.4E+00	0.53	5.70	500000	0.49	0.04
			T	379						
			C	1215	2.9E+00	0.46	5.63	430000		
			T	419						
			C	1322	3.0E+00	0.47	5.64	440000		
			T	447						
	750000	5.9	C	1478	3.8E+00	0.57	5.74	550000	0.64	0.07
			T	394						
			C	1438	5.2E+00	0.72	5.88	750000		
			T	275						
			C	1458	4.3E+00	0.64	5.80	635000		
			T	337						

Target Molecules (T)	Competitor Molecules (c)	log c		Pick Area (A)	Ac/AT	log(Ac/AT)	calculated X	calculated competitor molecules	Averages of log (C/T)	SD
Experiment A										
100 000	10000	4.0	C	87	7.9E-02	-1.10	3.96	9200	-1.03	0.072
			T	1096						
			C	62	9.3E-02	-1.03	4.03	10700		
			T	669						
			C	70	1.1E-01	-0.96	4.11	13000		
			T	630						
	50000	4.7	C						-0.35	0.022
			T							
			C	596	4.6E-01	-0.34	4.73	54000		
			T	980						
			C	402	4.3E-01	-0.37	4.70	50000		
			T	937						
	75000	4.9	C	490	6.7E-01	-0.18	4.89	76000	-0.21	0.028
			T	736						
			C	546	6.1E-01	-0.22	4.85	71000		
			T	899						
			C	546	5.9E-01	-0.23	4.83	68000		
			T	928						
	100000	5.0	C	635	9.7E-01	-0.01	5.05	112000	-0.05	0.043
			T	656						
			C	587	9.2E-01	-0.04	5.03	107000		
			T	637						
			C	647	8.0E-01	-0.10	4.97	94000		
			T	809						
	500000	5.7	C	4552	4.4E+00	0.64	5.71	510000	0.60	0.043
			T	1033						
			C	3825	3.9E+00	0.59	5.65	450000		
			T	987						
			C	4621	3.6E+00	0.56	5.62	420000		
			T	1273						
	750000	5.9	C	5472	7.6E+00	0.88	5.94	870000	0.81	0.099
			T	719						
			C	5132	5.0E+00	0.70	5.76	580000		
			T	1022						
			C	4826	7.3E+00	0.86	5.93	850000		
			T	661						

Target Molecules (T)	Competitor Molecules (c)	log c		Pick Area (A)	Ac/AT	log(Ac/AT)	calculated X	calculated competitor molecules	Averages of log (C/T)	SD
Experiment B										
100 000	10000	4.0	C	125	1.1E-01	-0.96	4.09	12200	-1.20	0.227
			T	1131						
			C	42	3.9E-02	-1.41	3.63	4300		
			T	1078						
			C	62	5.9E-02	-1.23	3.82	6600		
			T	1045						
	50000	4.7	C	1262	7.8E-01	-0.11	4.94	87000	-0.10	0.012
			T	1623						
			C	1506	8.2E-01	-0.09	4.96	91000		
			T	1841						
			C	1739	7.8E-01	-0.11	4.94	87000		
			T	2221						
	75000	4.9	C	1687	9.0E-01	-0.05	5.00	100000	-0.04	0.028
			T	1868						
			C	1575	8.7E-01	-0.06	4.98	95000		
			T	1807						
			C	1788	9.8E-01	-0.01	5.04	110000		
			T	1817						
	100000	5.0	C	2093	1.1E+00	0.04	5.09	123000	0.01	0.030
			T	1901						
			C	2430	9.8E-01	-0.01	5.04	110000		
			T	2473						
			C	1594	9.7E-01	-0.01	5.03	108000		
			T	1645						
	500000	5.7	C	2685	3.1E+00	0.49	5.53	340000	0.51	0.020
			T	876						
			C	1682	3.4E+00	0.53	5.57	370000		
			T	500						
			C	1646	3.2E+00	0.51	5.55	355000		
			T	509						
	750000	5.9	C	1748	4.8E+00	0.68	5.73	540000	0.71	0.057
			T	361						
			C	2544	6.0E+00	0.78	5.82	660000		
			T	427						
			C	1667	4.7E+00	0.67	5.71	515000		
			T	356						

Target Molecules (T)	Competitor Molecules (c)	log c		Pick Area (A)	Ac/AT	log(Ac/AT)	calculated X	calculated competitor molecules	Averages of log (C/T)	SD
Experiment C										
100 000	10000	4.0	C	86	5.4E-02	-1.27	3.72	5300	-1.23	0.031
			T	1771						
			C	104	6.0E-02	-1.22	3.77	5900		
			T	1740						
			C	109	6.2E-02	-1.21	3.78	6100		
			T	1759						
	50000	4.7	C						-0.41	0.018
			T							
			C	606	3.8E-01	-0.42	4.57	37500		
			T	1607						
			C	678	4.0E-01	-0.40	4.59	39000		
			T	1686						
	75000	4.9	C	1039	7.6E-01	-0.12	4.87	74000	-0.11	0.027
			T	1373						
			C	1094	8.3E-01	-0.08	4.91	82000		
			T	1313						
			C	971	7.4E-01	-0.13	4.86	73000		
			T	1321						
	100000	5.0	C	1858	1.1E+00	0.06	5.05	112000	-0.02	0.074
			T	1620						
			C	1784	8.2E-01	-0.09	4.91	82000		
			T	2171						
			C	1692	9.1E-01	-0.04	4.95	90000		
			T	1853						
	500000	5.7	C	1205	9.0E+00	0.95	5.95	890000	0.92	0.047
			T	134						
			C	1234	7.3E+00	0.86	5.85	710000		
			T	169						
			C	1180	8.5E+00	0.93	5.92	830000		
			T	139						
	750000	5.9	C	1316	1.4E+01	1.13	6.12	1310000	1.11	0.026
			T	97						
			C	1094	1.3E+01	1.12	6.11	1280000		
			T	83						
			C	1066	1.2E+01	1.08	6.07	1170000		
			T	88						

Target Molecules (T)	Competitor Molecules (c)	log c		Pick Area (A)	Ac/AT	log(Ac/AT)	calculated X	calculated competitor molecules	Averages of log (C/T)	SD
1 000 000	500000	5.7	C	404	3.3E-01	-0.48	5.59	390000	-0.40	0.11
			T	1229						
			C	596	5.3E-01	-0.27	5.80	630000		
			T	1118						
			C	455	3.7E-01	-0.44	5.63	430000		
			T	1242						
	1000000	6.0	C	860	9.4E-01	-0.03	6.04	1100000	0.00	0.04
			T	915						
			C	1010	1.1E+00	0.05	6.11	1300000		
			T	906						
			C	872	9.7E-01	-0.01	6.06	1150000		
			T	899						
	5000000	6.7	C	820	3.5E+00	0.54	6.61	4100000	0.54	0.03
			T	237						
			C	925	3.2E+00	0.51	6.58	3800000		
			T	285						
			C	922	3.7E+00	0.56	6.63	4300000		
			T	252						
	10000000	7.0	C	923	8.0E+00	0.90	6.97	9400000	0.96	0.07
			T	116						
			C	1133	8.9E+00	0.95	7.02	10400000		
			T	127						
			C	894	1.1E+01	1.03	7.10	12000000		
			T	83						

Target Molecules (T)	Competitor Molecules (c)	log c		Pick Area (A)	Ac/AT	log(Ac/AT)	calculated X	calculated competitor molecules	Averages of log (C/T)	SD
10 000	5000	3.7	C	439	6.2E-01	-0.20	3.84	7000	-0.38	0.15
			T	703						
			C	411	3.4E-01	-0.47	3.58	3800		
			T	1202						
			C	502	3.4E-01	-0.47	3.58	3800		
			T	1471						
	10000	4.0	C	585	1.1E+00	0.05	4.10	12500	0.03	0.02
			T	520						
			C	795	1.1E+00	0.04	4.08	12000		
			T	728						
			C	1140	1.0E+00	0.01	4.06	11500		
			T	1105						
	50000	4.7	C	1651	4.9E+00	0.69	4.73	54000	0.64	0.04
			T	340						
			C	1424	4.2E+00	0.62	4.67	47000		
			T	338						
			C	1636	4.0E+00	0.61	4.65	45000		
			T	405						
	75000	4.9	C	1642	5.7E+00	0.75	4.80	63500	0.70	0.05
			T	290						
			C	1772	4.6E+00	0.66	4.71	51500		
			T	384						
			C	1954	4.7E+00	0.67	4.71	51500		
			T	418						
	100000	5.0	C	1394	1.1E+01	1.03	5.07	118000	0.91	0.13
			T	130						
			C	1838	8.4E+00	0.93	4.97	94000		
			T	218						
			C	1752	6.0E+00	0.78	4.82	66000		
			T	294						
