# Supplementary material for: Competitive RT-PCR Strategy for Quantitative Evaluation of the Expression of Tilapia (Oreochromis niloticus) Growth Hormone Receptor Type I
Source: Biol Proced Online. 2009 Mar 10;11:79–98. doi: 10.1007/s12575-009-9002-3 (PMC3055623; doi:10.1007/s12575-009-9002-3)

Supplemental file 2

	Competitor molecules number, C	Log C		Pick area	C/T	log (C / T)	GHR I molecule number in the sample	Subunit 28S area	ug of total RNA in the RT reaction	GHR I molecules/ug total RNA	Average
**liver**											
Tilapia 1	300000	5.48	C	2709	7.09	0.85	50000	35	0.02	2500000	2055138
			T	382							
	100000	5.00	C	781	1.91	0.28					**SD***
			T	408							284756
	50000	4.70	C	237	1.05	0.02					
			T	225							**CV** (%)**
Tilapia 2	500000	5.70	C	3120	1.82	0.26	330000	541	0.19	1736842	15.89
			T	1713							
	200000	5.30	C	1904	0.63	-0.20					
			T	3024							
Tilapia 3	300000	5.48	C	1392	0.87	-0.06	300000	271	0.14	2142857	
			T	1591							
Tilapia 4	300000	5.48	C	1091	0.27	-0.57	800000	665	0.35	2285714	
			T	4014							
	600000	5.78	C	3887	0.74	-0.13					
			T	5218							
	800000	5.90	C	2264	1.01	0.00					
			T	2251							

* Standard deviation
** Coeficient of variation

	Competitor molecules number, C	Log C		Pick area	C/T	log (C / T)	GHR I molecule number in the sample	Subunit 28S area	ug of total RNA in the RT reaction	GHR I molecules/ug total RNA	Average
**spleen**											
Tilapia 1	500000	5.70	C	2973	10.08	1.00	50000	318	1.2	41667	77698
			T	295							**SD**
	200000	5.30	C	1566	3.87	0.59					39541
			T	405							
Tilapia 2	500000	5.70	C	1455	6.04	0.78	100000	2616	1.4	71429	**CV (%)**
			T	241							50.76
	200000	5.30	C	504	2.28	0.36					
			T	221							
Tilapia 3	300000	5.48	C	1625	1.03	0.01	300000	4850	2.5	120000	
			T	1577							

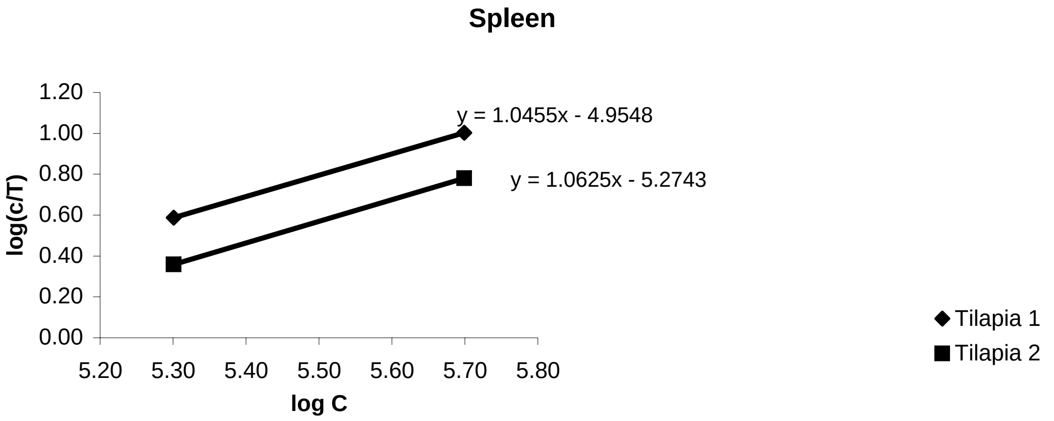

	Competitor molecules number, C	Log C		Pick area	C/T	log (C / T)	GHR I molecule number in the sample	Subunit 28S area	ug of total RNA in the RT reaction	GHR I molecules/ug total RNA	Average
**stomach**											
Tilapia 1	500000	5.70	C	7815	8.70	0.94	88000	2867	1.5	58667	94772
			T	898							
	200000	5.30	C	3891	2.91	0.46					**SD**
			T	1335							44437
Tilapia 2	500000	5.70	C	1640	5.62	0.75	130000	3064	1.6	81250	
			T	292							**CV (%)**
	200000	5.30	C	939	1.62	0.21					44.88
			T	580							
Tilapia 3	300000	5.48	C	3221	1.69	0.23	177000	2425	1.3	144400	
			T	1902							
	200000	5.30	C	864	1.14	0.06					
			T	755							

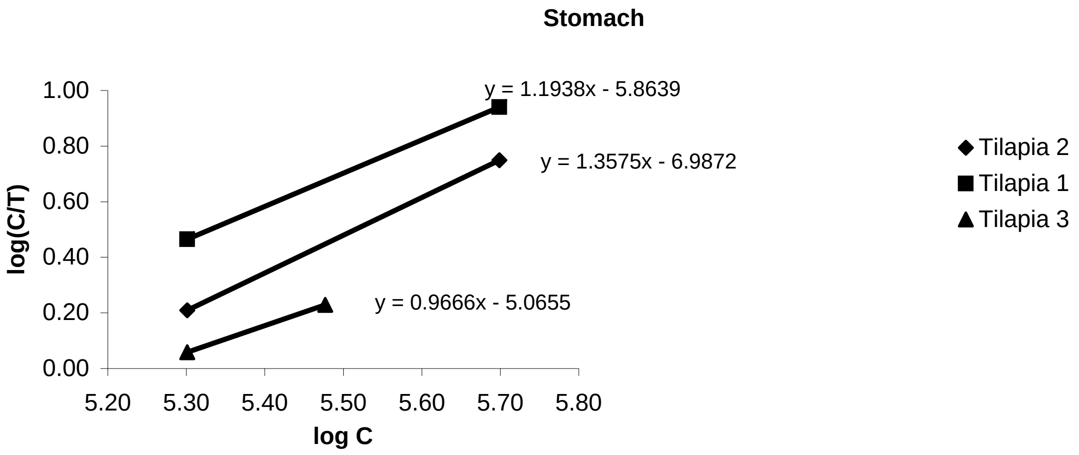

	Competitor molecules number, C	Log C		Pick area	C/T	log (C / T)	GHR I molecule number in the sample	Subunit 28S area	ug of total RNA in the RT reaction	GHR I molecules/ug total RNA	Average
**intestine**											
Tilapia 1	500000	5.70	C	327	1.04	0.02	500000	4326	2.25	222222	180773
			T	313							**SD**
Tilapia 2	500000	5.70	C	2306	2.57	0.41	280000	4320	2.25	124444	50559
			T	898							
	200000	5.30	C	932	0.77	-0.11					**CV (%)**
			T	1209							29.40
Tilapia 3	400000	5.60	C	1247	0.86	-0.07	450000	4416	2.3	195652	
			T	1457							
	300000	5.48	C	562	0.58	-0.24					
			T	974							

Intestine

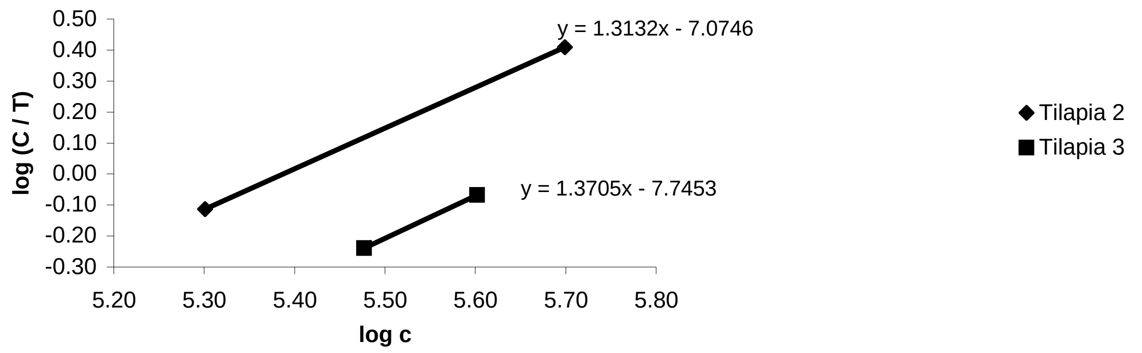

	Competitor molecules number, C	Log C		Pick area	C/T	log (C / T)	GHR I molecule number in the sample	Subunit 28S area	ug of total RNA in the RT reaction	GHR I molecules/ug total RNA	Average
**gonads**											
Tilapia 1	500000	5.70	C	4867	8.77	0.94	55000	478	0.25	220000	241935
			T	555							**SD**
	200000	5.30	C	1144	3.75	0.57					33092
			T	305							
Tilapia 2	500000	5.70	C	2259	0.77	-0.11	700000	5948	3.1	225806	**CV (%)**
			T	2930							13.21
	200000	5.30	C	248	0.28	-0.55					
			T	890							
Tilapia 3	500000	5.70	C	1490	0.83	-0.08	700000	4799	2.5	280000	
			T	1791							
	600000	5.78	C	244	0.98	-0.01					
			T	249							

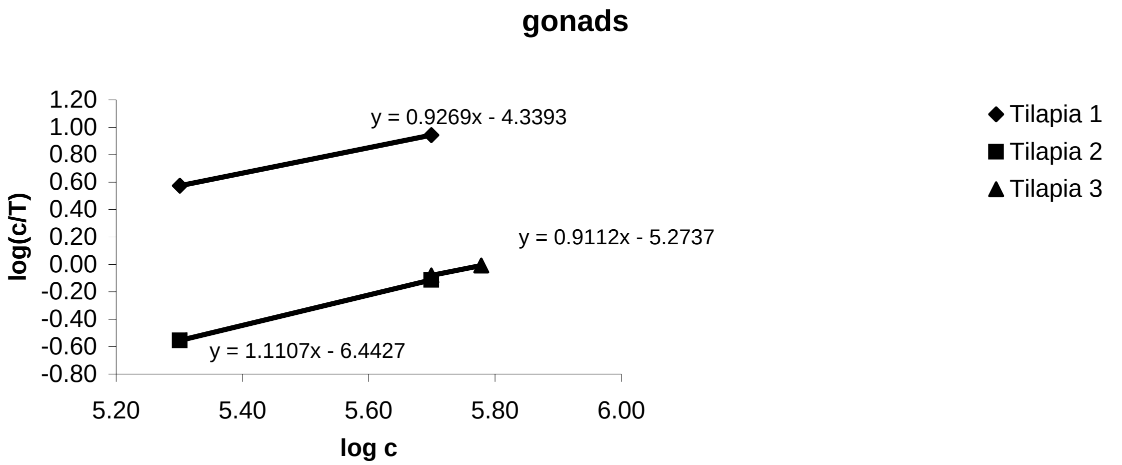

	Competitor molecules number, C	Log C		Pick area	C/T	log (C / T)	GHR I molecule number in the sample	Subunit 28S area	ug of total RNA in the RT reaction	GHR I molecules/ug total RNA	Average
**muscle**											
Tilapia 1	500000	5.70	C	9367	12.73	1.10	89000	255	0.13	684615	1563205
			T	736							**SD**
	300000	5.48	C	2511	6.51	0.81					983080
			T	386							
Tilapia 2	500000	5.70	C	2057	3.88	0.59	138000	196	0.1	1380000	**CV (%)**
			T	530							61.33
	300000	5.48	C	1623	2.32	0.36					
			T	701							
Tilapia 3	300000	5.48	C	1459	1.39	0.14	210000	150	0.08	2625000	
			T	1050							
	200000	5.30	C	1900	0.96	-0.02					
			T	1975							

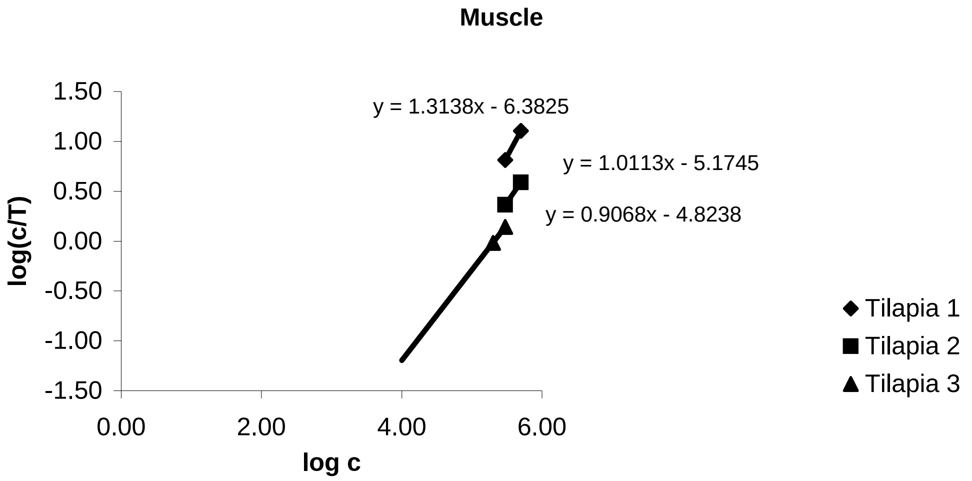

	Competitor molecules number, C	Log C		Pick area	C/T	log (C / T)	GHR I molecule number in the sample	Subunit 28S area	ug of total RNA in the RT reaction	GHR I molecules/ug total RNA	Average
**heart**								**3X**			
Tilapia 1	500000	5.70	C	3750	3.83	0.58	170000	2850	0.5	340000	377980
			T	979							**SD**
	200000	5.30	C	1050	1.23	0.09					77068
			T	851							
Tilapia 2	500000	5.70	C	1848	2.22	0.35	280000	3492	0.6	466667	**CV (%)**
			T	831							20.55
	300000	5.48	C	1366	1.23	0.09					
			T	1114							
Tilapia 3	300000	5.48	C	5409	4.02	0.60	108000	1886	0.33	327273	
			T	1346							
	100000	5.00	C	2873	0.91	-0.04					
			T	3157							

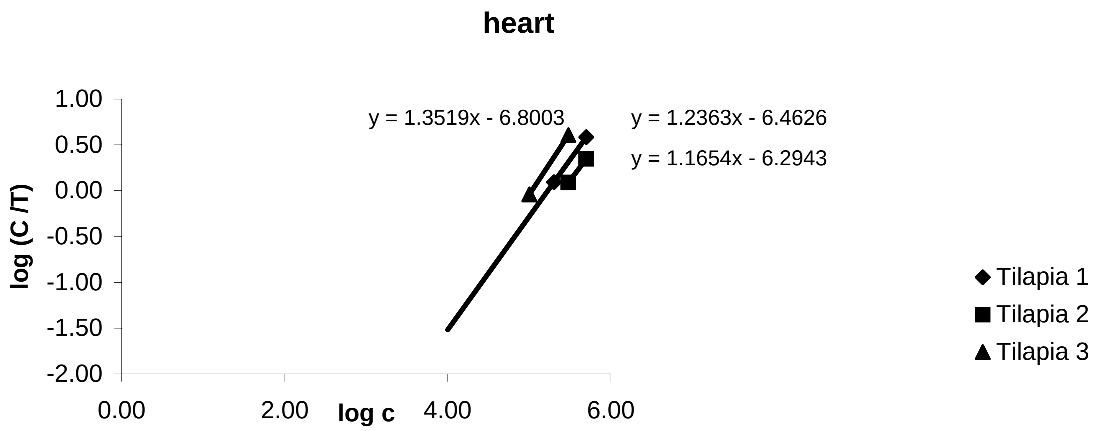

	Competitor molecules number, C	Log C		Pick area	C/T	log (C / T)	GHR I molecule number in the sample	Subunit 28S area	ug of total RNA in the RT reaction	GHR I molecules/ug total RNA	Average
**brain**											
Tilapia 1	500000	5.70	C	3587	2.55	0.41	206000	1541	0.27	762963	610608
			T	1406							**SD**
	200000	5.30	C	427	0.99	-0.01					420158
			T	432							
Tilapia 2	500000	5.70	C	2788	6.99	0.84	103000	4342	0.76	135526	**CV (%)**
			T	399							68.17
	200000	5.30	C	1812	2.27	0.36					
			T	800							
Tilapia 3	500000	5.70	C	15180	9.43	0.97	84000	547	0.09	933333	
			T	1610							
	200000	5.30	C	4938	3.00	0.48					
			T	1647							

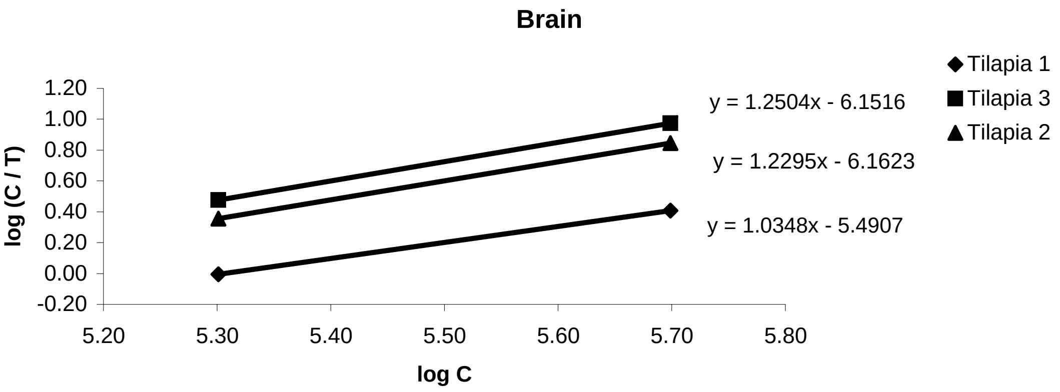

Supplement: Additional file 2 [file 1480-9222-11-1-9002-S2.pdf]
